# Disseminated Kaposi Sarcoma

**DOI:** 10.5811/cpcem.2021.9.53692

**Published:** 2021-11-01

**Authors:** Laura E. Goyack, Matthew A. Heimann

**Affiliations:** University of Alabama – Birmingham, Department of Emergency Medicine, Birmingham, Alabama

**Keywords:** Kaposi sarcoma, acquired immunodeficiency syndrome (AIDS), violaceous, adenopathy, point-of-care ultrasound (POCUS)

## Abstract

**Case Presentation:**

A 28-year-old male with a recent diagnosis of human immunodeficiency virus presented to the emergency department with odynophagia and dysphagia for a month. Physical exam revealed Kaposi sarcoma partially occluding the airway. Point-of-care ultrasound was used to assist with the diagnosis of reactive lymphadenopathy, and computed tomography revealed systemic disease. Otolaryngology was urgently consulted, and the patient was admitted for prompt tracheostomy the following day.

**Discussion:**

Kaposi sarcoma is a violaceous vascular neoplasm that is an acquired immuno-deficiency syndrome (AIDS)-defining illness. Mucocutaneous membranes should be thoroughly evaluated with patients suspected of AIDS. This case demonstrates the vital evaluation of the patient’s airway to assess patency. Highly active antiretroviral therapy should be initiated promptly, as well as chemotherapy in severe systemic cases.

## CASE PRESENTATION

**Image 1:**
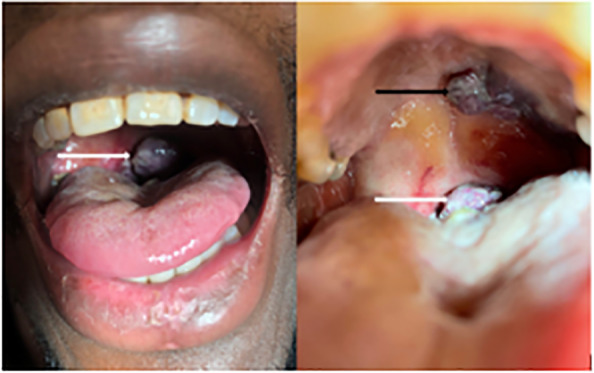
Midline posterior, pharyngeal pedunculated mass (white arrow) and hard palate mass (black arrow).

A 28-year-old male presented to the emergency department (ED) with progressively worsening odynophagia and dysphagia. He had been diagnosed with human immunodeficiency virus five months prior after initially presenting to an outside hospital with odynophagia and weight loss. Given difficulty affording his antiretrovirals, he reported only intermittently taking emtricitabine-tenofovir and was lost to follow-up. He was unaware of his oral lesions and overall grim clinical status. Seven days prior to presentation he developed mild dyspnea as well as worsening dysphagia. Physical examination of the oral cavity revealed a 3 × 3 centimeter (cm) violaceous, pedunculated, midline posterior mass partially occluding the airway, as well as a 2 × 2 cm anterior hard palate, violaceous mass (Image 1).

**Image 2:**
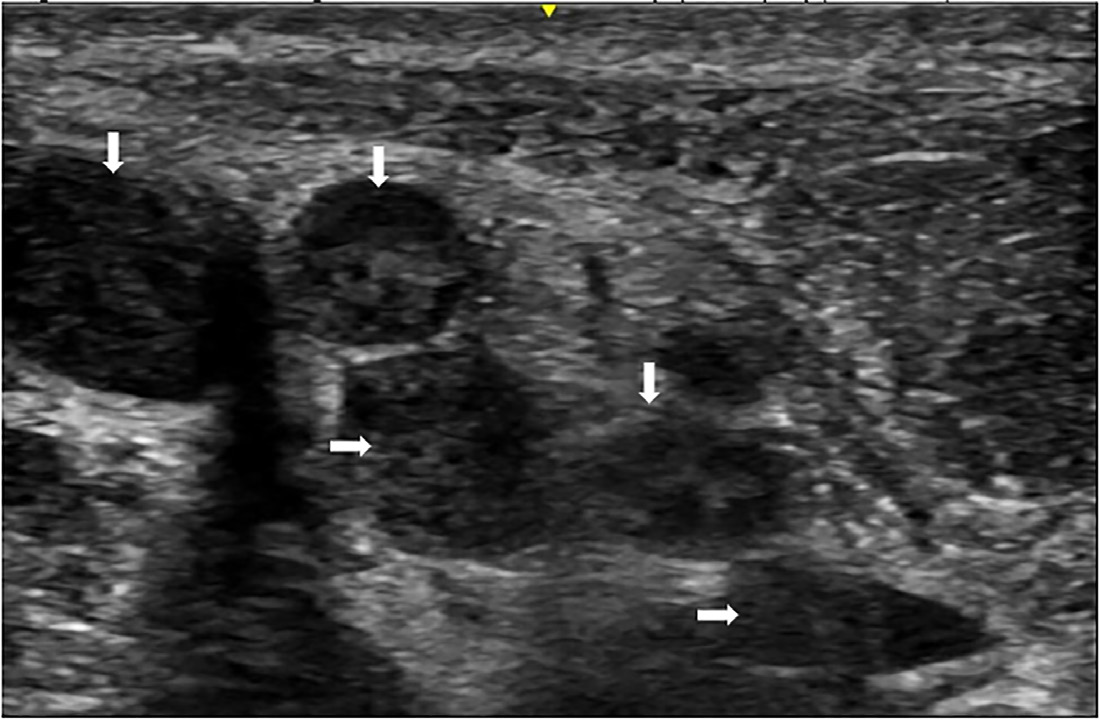
Point-of-care ultrasound images with extensive cervical reactive lymphadenopathy (white arrows).

The exam was also notable for extensive cervical and inguinal lymphadenopathy, global wasting, and a muffled voice. He was able to speak in full sentences, was tolerating his secretions, and did not have evidence of respiratory distress. Point-of-care ultrasound (POCUS) revealed reactive cervical lymphadenopathy (Image 2).

**Image 3:**
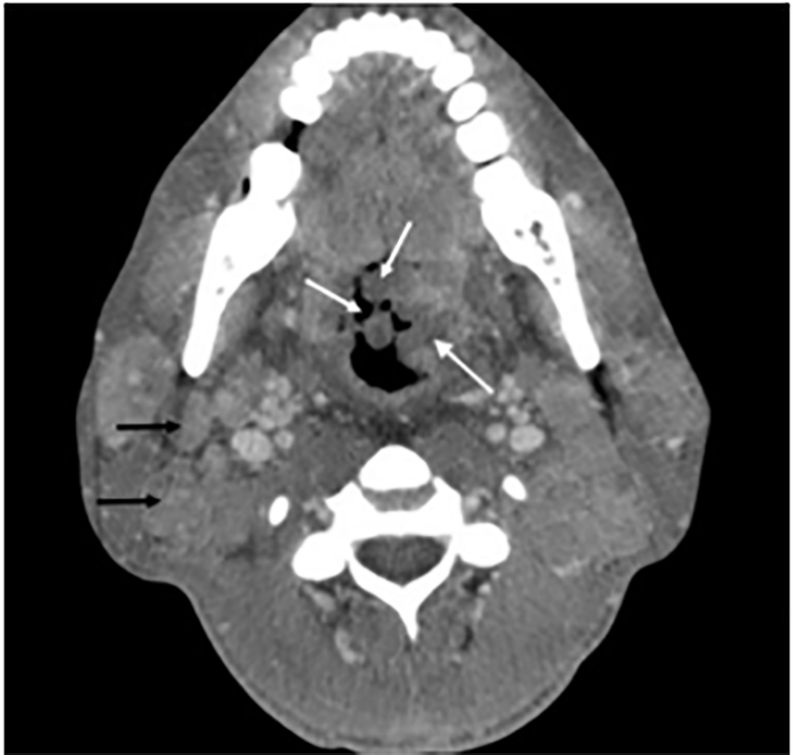
Axial computed tomography soft tissue neck showing diffuse nodular thickening of mucosal surfaces (white arrows) and extensive cervical lymphadenopathy (black arrows).

Computed tomography soft tissue neck (Image 3) and chest revealed nodular thickening of upper airway structures, cervical lymphadenopathy, diffuse ground-glass opacities, and large bilateral pleural effusions.

He was started on broad spectrum antibiotics including trimethoprim/sulfamethoxazole for *Pneumocystis jirovecii* pneumonia prophylaxis. Otolaryngology was consulted in the ED and performed a bedside laryngoscopy.. He was admitted for tracheostomy the following day. Excisional biopsy confirmed the diagnosis of disseminated Kaposi sarcoma. Given his extensive disease burden, he was started on systemic chemotherapy.

## DISCUSSION

Kaposi sarcoma traditionally occurs in patients with immunosuppression, such as those with acquired immunodeficiency syndrome (AIDS), or those who are immunosuppressed secondary to organ transplant. Kaposi sarcoma related to AIDS occurs in patients with cluster of differentiation four (CD4) counts less than 200 cells per cubic millimeter and is an AIDS-defining illness.^1^ Etiology is human herpesvirus-8, which causes endothelial cell proliferation leading to vascular neoplasia with multisystem involvement.^2^ Presentation includes erythematous or violaceous macules and plaques that progress to tumors or nodules.^2^ Lesions typically present at mucocutaneous sites, trunk, lower extremities, lymph nodes, lungs, and the gastrointestinal system. Diagnosis is made by history and physical examination revealing lesions and lymphadenopathy and is confirmed by tissue biopsy. Kaposi sarcoma responds to HIV suppression by highly active antiretroviral therapy (HAART). For severe systemic forms, chemotherapy can be combined with HAART.^3^

In our case, we used POCUS to confirm the diagnosis of extensive reactive lymphadenopathy. Other differentials included lymphoma, metastasis, abscess, and tuberculosis. Gray scale sonography can evaluate nodal morphology by noting size, shape, and architecture. Metastatic lymph nodes have loss of hilar architecture and the presence of intranodal calcification and necrosis.^4^ Using power Doppler, normal and reactive nodes will reveal hilar vascularity or will be avascular, while metastatic nodes will reveal peripheral or mixed vascularity.^5^

**CPC-EM Capsule**

What do we already know about this clinical entity?

*Kaposi sarcoma is an acquired immunodeficiency syndrome-defining illness.*

What is the major impact of the image(s)?

*Few images of Kaposi sarcoma of the oral cavity exist. This multimodality image approach illustrates the pathology and severity of the illness beyond cutaneous findings.*

How might this improve emergency medicine practice?

*Thorough physical examination and multimodal imaging could aid the emergency physician in making this life-altering diagnosis.*
